# Information and Communication Technologies in Patients With Immune-Mediated Inflammatory Diseases: Cross-sectional Survey

**DOI:** 10.2196/37445

**Published:** 2022-09-13

**Authors:** Esther Chamorro-de-Vega, Rosa Romero-Jiménez, Vicente Escudero-Vilaplana, Arantza Ais-Larisgoitia, María Elena Lobato Matilla, Carlos M González, Luis Menchén, Ofelia Baniandrés, Lucía Ibares-Frias, Carmen Lobo-Rodríguez, Ana Herranz-Alonso, María Sanjurjo

**Affiliations:** 1 Hospital General Universitario Gregorio Marañón Instituto de Investigación Sanitaria Gregorio Marañón Madrid Spain; 2 Department of Pharmacy Hospital General Universitario Gregorio Marañón Instituto de Investigación Sanitaria Gregorio Marañón Madrid Spain; 3 Department of Rheumatology Hospital General Universitario Gregorio Marañón Instituto de Investigación Sanitaria Gregorio Marañón Madrid Spain; 4 Department of Gastroenterology Hospital General Universitario Gregorio Marañón Instituto de Investigación Sanitaria Gregorio Marañón Madrid Spain; 5 Departamento de Medicina Facultad de Medicina Universidad Complutense Madrid Spain; 6 Department of Dermatology Hospital General Universitario Gregorio Marañón Instituto de Investigación Sanitaria Gregorio Marañón Madrid Spain; 7 Department of Ophthalmology Hospital General Universitario Gregorio Marañón Instituto de Investigación Sanitaria Gregorio Marañón Madrid Spain; 8 Nursing Group Hospital General Universitario Gregorio Marañón Instituto de Investigación Sanitaria Gregorio Marañón Madrid Spain

**Keywords:** mHealth, app, information and communication technologies, immune-mediated inflammatory diseases, IMID, cross-sectional survey, survey, monitoring, clinical support, clinicians, quality of care, patient care, mobile app, tool, management tool

## Abstract

**Background:**

Information and communication technologies (ICTs) are changing the traditional health care model and redefining personalized health. ICTs offer effective communication and real-time monitoring of patients and provide additional data to support clinical decision-making, improve the quality of care, and contribute to the empowerment of patients. However, evidence on the use of ICTs and digital preferences of immune-mediated inflammatory disease (IMID) patients is scarce.

**Objective:**

The aim of this study is to describe the degree of use of ICTs in patients with IMIDs (including rheumatic diseases, inflammatory bowel diseases, and psoriasis), identify their needs, and analyze their interest in the use of apps as tools for better management of their disease.

**Methods:**

A questionnaire was created by a multidisciplinary team including pharmacists, rheumatologists, gastroenterologists, dermatologists, and nurses with experience in ICTs applied to the field of IMID. The survey included 27 questions organized into 3 blocks: (1) sociodemographic characteristics, (2) ICT use for health-related information, and (3) patient expectations about mobile health.

**Results:**

A total of 472 questionnaires were analyzed. Overall, 52.9% (250/472) of patients were diagnosed with a rheumatologic disease, 39.4% (186/472) with inflammatory bowel disease, and 12.3% (58/472) with psoriasis. The state of health was considered good by 45.6% (215/472) of patients. Patients were interested in staying informed about health issues in 86.9% (410/427) of cases and sought health-related information mainly from the internet (334/472, 70.8%) and health care professionals (318/472, 67.4%). Overall, 13.6% (64/472) did not trust the health information they found in internet. Of the patients, 42.8% (202/472) had a health app, and 42.2% (199/472) had found it on their own. Patients would like a health app to help mainly to manage appointments (281/472, 59.5%), obtain information about their diseases and treatments (274/472, 58.1%), and get in contact with health professionals (250/472, 53.0%). Overall, 90.0% (425/472) of patients reported they would use an app to manage their IMID if their health professional recommended it, and 58.0% (274/472) would pay or probably be willing to pay for it.

**Conclusions:**

IMID patients were very interested in finding health-related information via ICTs, especially using smartphones and apps recommended by health professionals. Appointment management, advice on disease and treatment management, and personalized communication with health professionals were the most desired app features identified. Health professionals should play an essential role in recommending and validating these tools to ensure they are of high quality.

## Introduction

Immune-mediated inflammatory diseases (IMID) are a large group of chronic diseases of high prevalence and a high socio-health impact [1]. Patients with IMID are generally middle age, with diverse clinical manifestations, and reduced quality of life what makes its handling complex [2,3]. These patients often report a lack of understanding of their disease, demand information in real time, and sometimes require unscheduled health care due to clinical decompensations, causing a high consumption of health care resources [4-6]. This situation represents a challenge for the usual structure of health care services, making it necessary to develop new models of clinical management that allow making intensive and regular disease monitoring as well as maintaining the sustainability of the system.

The recent emergence of information technologies (ICTs), concretely mobile health (mHealth), has been positioned as key tools to improve the quality of health care since it helps both professionals and patients. On the one hand, ICTs allow health care professionals to access the patient’s clinical information in real time, improving risk monitoring and enabling proactive treatment. On the other hand, these tools facilitate connected care and allow patients to have direct communication with their health professionals [7-11]. Moreover, ICTs favored the conciliation of patients with other activities of their daily life, promoting their autonomy, and streamlining processes and bureaucratic procedures. Thus, ICTs, especially apps, have the potential for promoting self-management, improving standard clinical care, and reducing the impact of the growing burden of IMID on health care resource utilization [12-15].

There is a wide variety of apps for IMID patients on the market with numerous features and functionalities but a lack of validity and scientific reliability [12,14,16]. The lack of regulation of these tools makes health care professionals primarily responsible for ensuring the validity of the tools they recommend to their patients. However, information and evidence about the real use of ICTs in IMID patients and the needs and expectations patients have for these technologies are scarce. This is essential for further validation and improvement of health-related app, from both a clinical and patient perspective [17-19]. With this study, we aimed to describe the degree of use of information and communication technologies in patients with IMID, identify their needs, and analyze their interest in the use of apps as a tool for better management of their disease.

## Methods

### Study Design and Setting

We performed a descriptive study in a tertiary care teaching hospital of the Madrid Public Health Service (Spain). This hospital has a coverage of the entire portfolio of services for a population of 325,000 inhabitants distributed in 11 basic health areas. This hospital has a comprehensive care center, a reference center for the management of IMID care, for patients with IMID.

### Study Patients

Patients diagnosed with IMID, including rheumatic disorders, inflammatory bowel disease, and psoriasis and treated with biological or targeted therapies, were eligible for the study. Patients under 18 years, those treated in a clinical trial, and those who had trouble understanding the questions due to language or cultural barriers were excluded. All patients provided written informed consent and agreed to participate.

### Data Collection

Data were collected from December 2020 to July 2021 during a single visit through an anonymous patient self-questionnaire. To design the questionnaire, the survey created by Spanish National Observatory of Telecommunications and Information Society [20] was taken as a model and modified and adapted to our population by a multidisciplinary team including pharmacists, rheumatologists, gastroenterologists, dermatologists, and nurses with experience in ICTs applied to the field of IMID. Finally, a 27-item questionnaire was designed (Multimedia Appendix 1). The survey comprised 3 main blocks. Block I: patient sociodemographic characteristics (questions 1-6); block II: ICT use for health-related information (questions 7-17); and block III: patient expectations about mHealth (questions 18-27). With respect to the types of questions, the questionnaire comprised dichotomous statements (2, 10, 15-18, 20, 25, and 26) with true or false response options or polytomous statements (3-9, 11-14, 19, and 21-24) categorical variables. Some statements were multiple choice (5, 7-9, 11-13, 21-24). Ten patients completed a draft paper questionnaire to validate the fact that it was well understood. Regarding the question of whether patients would be willing to pay to download an app, the cost of the app was taken from the review published by Collado-Borrell et al [21] who showed an average price of apps for patients with cancer of 2.15€ (US $2.15).

A paper copy of the questionnaire was handed to every patient in the infusion center, in pharmaceutical consultation, or in the waiting rooms of the comprehensive care center. Alternatively, patients could take the questionnaire home and return the completed form at their next appointment.

### Statistical Analysis

Data were analyzed using SPSS for Windows (version 21.0, IBM Corp) software. Variables were analyzed using descriptive statistics. Normality was analyzed by means of the Kolmogorov-Smirnov test. Numeric variables were compared with the Student *t* test or the Mann-Whitney test. The association between qualitative variables was studied using the Pearson chi-square test or Fisher exact test. The corresponding measures of association and risk were calculated along with their confidence intervals. Results with a value of *P*<.05 were considered statistically significant.

### Ethics Approval

The study protocol was approved by the Ethics Committee of the Hospital General Universitario Gregorio Marañón (approval number: IMID-HGUGM.01), in accordance with the principles of the Declaration of Helsinki. The questionnaire did not include any information about the personal data of patients to ensure data confidentiality.

## Results

### Patient Sociodemographic Characteristics

Of the questionnaires distributed, there was a participation of 95.2% (472/496). The remaining 24 questionnaires were not analyzed because they were not understandable. Patient sociodemographic characteristics are shown in Table 1. The median age of patients was 50.5 years (IQR 40.0-59.8). Of all participants, 53.2% (251/472) were women and 44.7% (211/472) had a university education.

Overall, 52.9% (250/472) of patients were diagnosed with a rheumatologic disease, 21.8% (103/472) rheumatoid arthritis, 16.7% (79/472) spondylarthritis, 13.1% (62/472) psoriatic arthritis, and 1.3% (6/472) other. Among nonrheumatologic patients, 39.4% (186/472) were inflammatory bowel disease, 71.5% (133/186) were Crohn disease, 28.5% (53/186) were ulcerative colitis, and 12.3% (58/472) were psoriasis. Overall, 12.1% (57/472) of patients were diagnosed with more than one concomitant IMID. Over half (269/472, 57.0%) of patients considered their state of health was either good 45.6% (215/472) or very good 11.4% (54/472).

**Table 1:** Patient sociodemographic characteristics (n=472).

Demographic characteristics		Value
**Sex, n (%)**		
	Female	251 (53.2)
	Male	221 (46.8)
**Age (years), mean (SD)**		49.9 (13.4)
	≤35, n (%)	74 (15.7)
	36-50, n (%)	159 (33.7)
	51-60, n (%)	128 (27.1)
	≥61, n (%)	106 (22.4)
	No response, n (%)	5 (1.1)
**Current occupation, n (%)**		
	Works	280 (59.3)
	Retired	88 (18.6)
	Unemployed	53 (11.2)
	Homemaker	35 (7.4)
	Study	15 (3.2)
	No response	1 (0.2)
**Level of education, n (%)**		
	No education or incomplete primary education	10 (2.1)
	Primary education	80 (16.9)
	Secondary education	167 (35.4)
	University education	211 (44.7)
	No response	4 (0.8)
**Type of IMID^a,b^, n (%)**		
	Crohn disease	133 (28.2)
	Rheumatoid arthritis	103 (21.8)
	Spondyloarthritis	79 (16.7)
	Psoriatic arthritis	62 (13.1)
	Psoriasis	58 (12.3)
	Ulcerative colitis	53 (11.2)
	Other	48 (10.2)
	More than one IMID	57 (12.1)
**How would you rate your overall health? n (%)**		
	Very good	54 (11.4)
	Good	215 (45.6)
	Average	172 (36.4)
	Poor	27 (5.7)
	Very poor	1 (0.2)
	No response	3 (0.6)

^a^Multiple choice question.

^b^IMID: immune-mediated inflammatory disease.

### ICT Use for Health-Related Information

**Figure 1:**
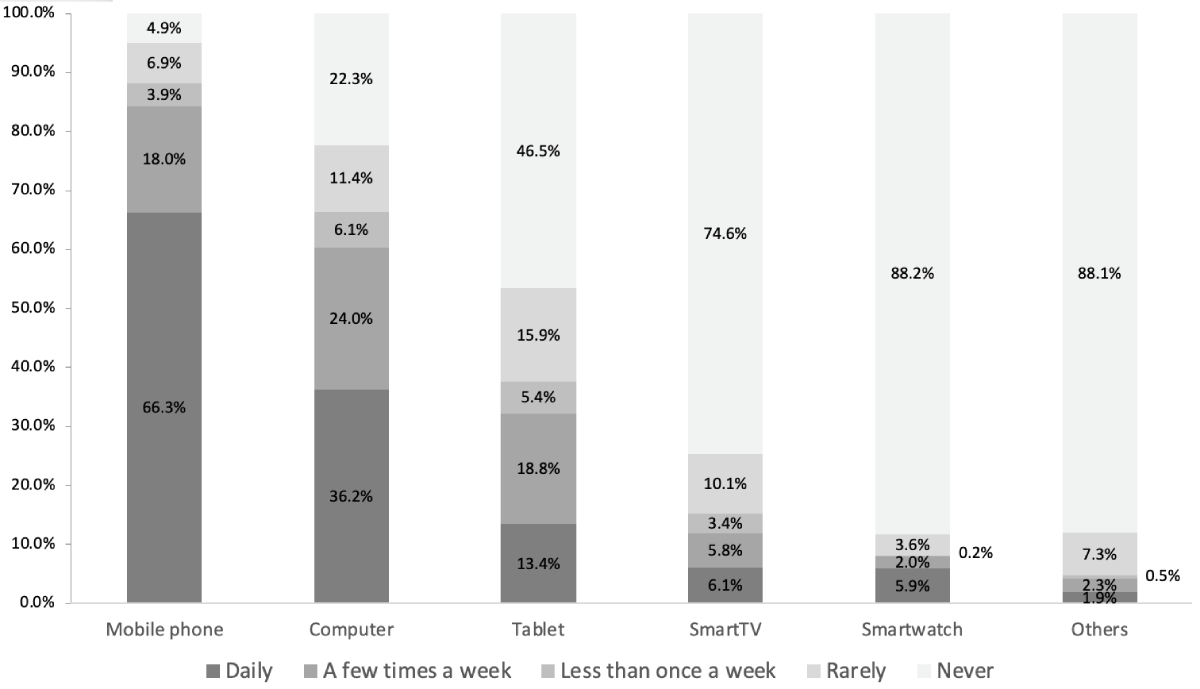
Frequency with which patients used different information and communication technologies to search for information on the internet.

Patients were questioned concerning internet use to address their habits regarding information search (Table 2). Most of the patients had heard the term app and smartphone (359/472, 76.1%, and 347/472, 73.5, respectively) before, but only 17.6% (83/472) had heard the term wearable. Mobile phones and desktops were the devices most used for searching for information on the internet (433/472, 91.7%, and 308/472, 65.3%, respectively). The mobile phone was used daily to search for information on the internet by 65.5% (309/472) of patients. Figure 1 shows the frequency with which patients used different ICTs to search for information on the internet.

Patients sought health-related information mainly from the internet and health care professionals (334/472, 70.8%, and 318/472, 67.4%, respectively). Of patients who consulted the internet, 73.5% (347/472) searched through Google and 33.3% (157/472) through social networks (YouTube, Twitter, and Facebook, among others) and 24.2% (114/472) used medical societies. About half of patients looked for health information to obtain information about disease prevention, healthy lifestyles, and health care (239/472, 50.6%) and to find information about the treatment their doctor had prescribed for them (215/472, 45.6%).

We observed statistically significant differences depending on the level of education, age, sex, and IMID type. First, patients with secondary or university education and younger patients searched for more information on health in the internet (*P*<.001). Patients with secondary or university education sought more information about health centers or health professionals (*P*<.001), regarding symptoms and learning about potential diseases (*P*=.003), and regarding their treatment (*P*=.02). Men sought more information regarding symptoms and learning about potential diseases (*P*=.02), while women wanted to get in contact with other people with similar health problems (*P*=.004). Patients with ulcerative colitis were most likely to seek information about their treatment (*P*=.02).

Regarding understanding internet health information, patients replied that it seemed easy always (49/472, 10.4%) or sometimes (113/472, 23.9%). For the question as to whether participants trusted the information they found on the internet, overall, 13.6% (64/472) responded no, but it was a statistically more frequent reply among young patients (*P*<.001) and homemakers and retired people had less trust than others (*P*<.001). Patients with secondary or university education reported more frequently than other groups that they trusted the information they found on the internet depending on the website (*P*<.001).

Overall, 16.7% (79/472) of patients turned to the internet for information about their disease or treatment before their doctor’s appointment, an action that was more common in younger patients (*P*=.02) and in students (*P*=.049). After doctor appointment, 18.8% (89/472) of patients always referred to the internet and 31.6% (150/472) only when they had doubts, being more frequent among student patients (*P*=.003) and those with secondary or university education (*P*=.002).

**Table 2:** Information technology use for health-related information (n=472).

Use of information and communication technologies			Value, n (%)
**Which of the following terms have you heard before?^a^**			
	App	359 (76.1)	
	Smartphone	347 (73.5)	
	Information and communication technologies	178 (37.7)	
	eHealth	111 (23.5)	
	Mobile health	89 (18.9)	
	Wearable	83 (17.6)	
	I have not heard any of these terms before	53 (11.2)	
	No response	6 (1.3)	
**Which devices do you use to look for information on the internet?^a^**			
	Mobile phone	433 (91.7)	
	Desktop or laptop computer	308 (65.3)	
	Tablet	199 (42.2)	
	Television with internet (SmartTV)	79 (16.7)	
	Smartwatch	34 (7.2)	
	Other	5 (1.1)	
	I do not look for information on the internet	17 (3.6)	
	No response	3 (0.6)	
**Are you interested in staying informed about health-related matters?**			
	Yes	410 (86.9)	
	No	36 (7.6)	
	No response	26 (5.5)	
**Where do you search for information on health?^a^**			
	Internet	334 (70.8)	
	Health professionals	318 (67.4)	
	People close to me (friends, relatives, workmates, etc)	116 (24.6)	
	Newspapers, magazines, pamphlets	89 (18.9)	
	Apps	71 (15.0)	
	Other	12 (2.5)	
	No response	27 (5.7)	
**If you use the internet to search for health information, which types of website do you use?^a^**			
	Google	347 (73.5)	
	Medical societies	114 (24.2)	
	Patient associations	87 (18.4)	
	YouTube	72 (15.3)	
	I do not search for medical information on the internet	64 (13.6)	
	Facebook	49 (10.4)	
	Blogs	37 (7.8)	
	Twitter	27 (5.7)	
	Other	15 (3.2)	
	Other social networks	10 (2.1)	
	No response	29 (6.1)	
**For what purposes do you search for health information?^a^**			
	Disease prevention, healthy lifestyle, health care	239 (50.6)	
	To find information about the treatment prescribed by my doctor	215 (45.6)	
	To find symptoms and learn about potential diseases	132 (28.0)	
	To find information about medical centers or health professionals	126 (26.7)	
	To find information about alternative/complementary medicines (herbal products, acupuncture, etc)	65 (13.8)	
	To get in contact with other people with health problems like mine	65 (13.8)	
	Other	21 (4.4)	
	No response	67 (14.2)	
**Is it easy to understand the health information you find on the internet?**			
	Usually	232 (49.1)	
	Sometimes	113 (23.9)	
	Always	49 (10.4)	
	Never	20 (4.2)	
	No response	58 (12.3)	
**Do you trust the health information you find on the internet?**			
	Depends on the website	321 (68.0)	
	No	64 (13.6)	
	Yes	43 (9.1)	
	No response	44 (9.3)	
**Do you look up information on the internet about your disease or treatment BEFORE going to your doctor’s appointment?**			
	No	367 (77.8)	
	Yes	79 (16.7)	
	No response	26 (5.5)	
**Do you look up information on the internet about your disease or treatment AFTER going to your doctor’s appointment?**			
	No	210 (44.5)	
	Only if I have still got doubts about something	150 (31.8)	
	Yes	89 (18.8)	
	No response	23 (4.9)	

^a^Multiple choice question.

### Patient Expectations About mHealth

Table 3 shows the results for preferences regarding the use of mHealth. Overall, 92.2% (435/472) of patients had a smartphone, and 42.8% (202/472) had installed at least 1 health app. We observed that most (199/472) of the patients had found the health-related apps for their will. Concerning mobile phone use, 73.9% (349/472) of patients used them to access the internet, and 66.5% (314/472) to use apps, among other functions.

**Figure 2:**
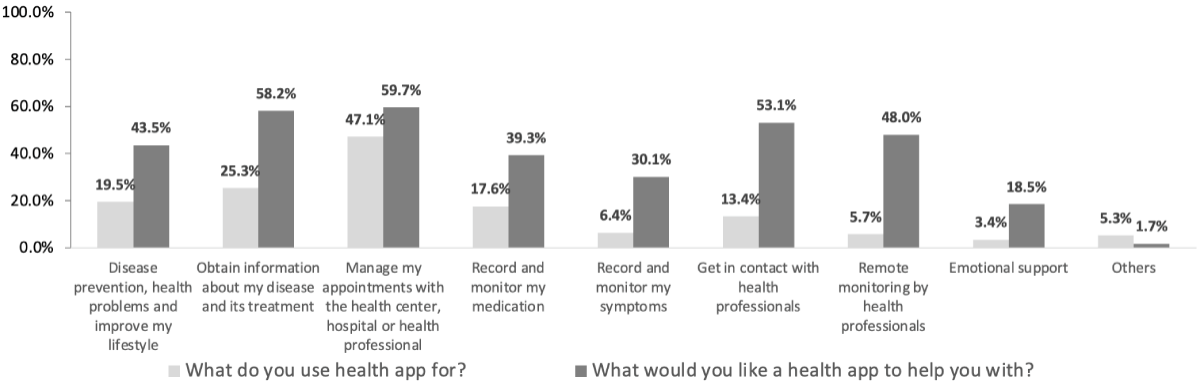
Reasons why patients used a health app, and things they wish the app could help them with.

Figure 2 shows the reasons why our patients used health apps and their use preferences. We found that patients used health-related apps mainly to manage appointments with health centers, hospitals, or health professionals (222/472, 47.0%) and to obtain information about diseases and treatments (119/472, 25.2%).

We found that younger patients used apps to obtain information about disease prevention, health problems, and improve their lifestyle (*P*=.05) and to record and monitor their symptoms (*P*=.01) with a greater frequency than the other sociodemographic groups. Patients with secondary or university education were statistically more inclined than other patient groups to use apps to record and monitor their symptoms (*P*<.04), record their medication (*P*=.04), and manage their appointments (*P*=.03). Unemployed patients and homemakers used apps more than those in other occupations to get in contact with other patients (*P*=.03). Furthermore, women used apps to obtain emotional support more frequently than men (*P*=.02).

When ask patients about what they would like a health app to help, most of them answered to manage appointments (281/472, 59.5%), to obtain information about their diseases and treatments (274/472, 58.1%), to get in contact with health professionals (250/472, 53.0%), and for remote monitoring by health professionals (226/472, 47.9%). We observed that women than men would like to use apps more to get emotional support (*P*=.001).

Overall, 90.0% (425/472) of patients reported they would use an app to manage their IMID if their health professionals recommended it, and 58.0% (274/472) patients would pay or probably be willing to pay for it, being more frequent in patients with secondary or university education (*P*<.001).

**Table 3:** Use preferences for health apps (n=472).

Use preferences for health apps		Value, n (%)
**Is your mobile phone a smartphone?**		
	Yes	435 (92.2)
	No	15 (3.2)
	No response	22 (4.7)
**Do you have health-related apps installed?**		
	No	250 (53.0)
	Yes	202 (42.8)
	No response	20 (4.2)
**How did you find the health-related apps you use?^a^**		
	Prescription or medical advice	76 (16.1)
	On my own	199 (42.2)
	On the recommendation of a family member or friend	73 (15.5)
	No response	180 (38.1)
**What do you use your mobile phone for?^a^**		
	Normal phone use (calls, messages, photos/videos, etc)	448 (94.9)
	To access the internet	349 (73.9)
	Schedule planner and alarms	323 (68.4)
	To use apps	314 (66.5)
	Social networks	245 (51.9)
	No response	19 (4.0)
**How would you like to communicate with your health professional?^a^**		
	Telephone	353 (75.0)
	Videoconference	202 (42.8)
	Email	201 (42.6)
	Apps	179 (37.9)
	Website	25 (5.3)
	Blogs	4 (0.8)
	Social networks	16 (3.4)
	No response	36 (7.6)
**Would you use an app if your health professional recommended it?**		
	No	21 (4.4)
	Yes	425 (90.0)
	No response	26 (5.5)
**Would you download a health-related app if you had to pay approximately €2.15 (US $2.15)?**		
	No	171 (36.2)
	Probably	174 (36.9)
	Yes	100 (21.2)
	No response	27 (5.7)

^a^Multiple choice question.

## Discussion

### Principal Findings

We have analyzed the needs and interests of a large cohort of IMID patients in ICTs as a means of managing their disease. We found that the most frequent ICTs used by far was the mobile phone (91.7%) and the most frequent source of information was the internet (70.8%; mainly in Google), ahead of health care professionals.

We showed that patients searched for health information on the internet mainly for disease prevention, healthy lifestyles, health care, and treatments prescribed by their doctors. Most (67.9%) of the patients trusted the information they found on the internet but that depended on the website.

Regarding the use of health apps, 42.8% of patients had health-related apps installed on their mobile phones, with only 16.1% found by medical advice. The most interesting features that patients expected to find in an app were appointment management, advice on disease and treatment management, and personalized communication with health professionals. Most (425/472, 90.0%) of the patients would use an app if their health professional recommended it and more than half (274/472, 58.1%) of patients would be willing to pay for it.

### Comparison With Prior Work

#### Patients Preferences in the Use of Apps

Digitization of health care and ICT has gained momentum in recent years, mainly sparked by the COVID-19 pandemic [22], changing the practice of medicine and the way in which health information about health and manage diseases is accessed [20,23,24]. However, data published about the use of mHealth technologies in IMID patients is focused on interventions regardless of the patient’s perspective, which is needed for further validation and improvement of these technologies [9-11,13,15]. To the best of our knowledge, this is the first survey to identify the frequency of use and needs and interests in ICTs as a mean of managing their disease in a multidisciplinary cohort of IMID patients, including patients with rheumatology disease, inflammatory bowel diseases, and psoriasis among others, carried out in a comprehensive care center for these patients. The characteristics of the questionnaire, which contains transversal questions, facilitate its extrapolation to other pathologies. Our colleagues had used a very similar questionnaire in hematology-oncology patients to understand the ICT use profile and identify their needs and interest [25].

#### ICT Use for Health-Related Information

The way in which people access health information is changing. As observed by Knitza et al [18], the internet was the most frequent source of health information, ahead of health care professionals (70.8% vs 67.4%), contrary to what is traditionally described, where more patients have tried to physically obtain medical information from ordinary health professionals [20,25,26]. Regarding the reasons why patients seek health information, most of the patients showed interest in disease prevention (50.5%) and in their treatments (45.6%), although this result was lower compared with other authors (67%-80%) [17,18]. A considerable percentage of patients (47.4%, 35.0%, and 13.8% reported by Magnol et al [17], Knitza et al [18], and in this study, respectively) seek information to get in contact with other people with similar problems. However, when patients were asked about what they would like a health app to do, they showed less interest in getting emotional support or direct exchange such as chats with peers with the same disease [18]. Health professionals can encourage patients to enroll in patient associations for a holistic approach to managing their condition due to its beneficial effects [27,28], and apps could be the platform for it.

The high complexity of managing IMIDs and their treatments, as well as the limited health care resources in many cases, causes patients to have many doubts that are not resolved by health professionals. In our study, more than half (239/472, 50.6%) of the patients consulted the internet after a medical visit. Knitza et al [18] showed that 75% of patients had previously used the internet to obtain health information during the last 3 months prior to the clinical visit. The internet can be an unreliable source of information if they don’t have the ability to make critical use of it [20,26,29]. In our study, only a small percentage of the patients found the information on the internet always easy to understand (10.4%). In addition, 13.4% did not trust the information they found. This highlights the need to establish legislation that regulates this aspect and the role of health professionals to provide guidance on where and how to look for health information [21,30].

#### Patient Expectations About mHealth

Regarding the use of mHealth, we found that 42.8% of our patients used health-related apps, a higher rate compared with 21.6% showed by Magnol et al [17] or 4.1% reported by Knitza et al [18] in rheumatology patients. This difference could be explained by the younger age of the patients included in our study or the heterogeneity of our patients with different IMIDs. Other chronic diseases has been associated with higher eHealth use [31-33]. In our study, most of the patients (42.2%) found the app on their own. This highlights once again the importance of health professionals in advising on the use of ICTs.

In general, the main interests of patients with apps were information about medications and diseases. Regarding the communication with health care professionals, our patients preferred the more traditional means of communication such as telephone, videoconferencing, and email, as also reported by Knitza et al [18], who observed that patients preferred to receive medical information on paper. However, we showed that one of the app functions that patients would like the most was to get in contact with health professionals (53.0%) and for remote monitoring by health professionals (47.9%). Moreover, 37.9% of patients would use an app as a means of communication with health care professionals. Finally, 90% of our patients would use an app to manage their IMID if their health professionals recommended it, a higher result than showed by Magnol et al [17] (69.9%). The widespread access and use of the mobile phone by patients can help health care professionals increase patient monitoring while improving patient convenience, especially for those who are functionally incapacitated or who live far away [34,35]. This fact, along with the significant demonstrated benefits of apps in terms of clinical care [12,33,36,37], cost-effectiveness [13,38], and versatility of its features (such as including patient-reported outcomes) [39-41], make apps a perfect tool for complement and improve the standard clinical care of patients with IMID.

### Limitations and Strengths

Our study has several limitations related to the characteristics of studies under real clinical practice. First, our study is limited by its single-center design. Consequently, these results might not be obtained in centers or countries with different characteristics. Second, the cross-sectional design, self-reported data, and sampling method can affect extrapolation of the results. However, a strength of this study is its multidisciplinary design with a systematic inclusion of large numbers of consecutive patients with different IMIDs, including rheumatology diseases, inflammatory bowel disease, and psoriasis among others, which resulted in a representative sample of patients with IMID. Third, there is great variability in the price of health-related app that can influence the response of patients to these new technologies depending on the cost indicated in the survey. A review showed that most apps were free, and specifically 82.2% of apps for IMID patients were free [42]. However, in IMID apps requiring payment, the cost is slightly higher (€9.10, or US $9.10) than the average observed in other reviews (€0.90-4.20, or US $0.90-4.20) [42]. Our patients are willing to pay for a health app if their health care professional recommends it.

### Conclusion

IMID patients were very interested in finding health-related information via ICTs, especially using smartphones and apps recommended by health professionals. We could successfully identify unmet needs and patient priorities, with appointment management, advice on disease and treatment management, and personalized communication with health professionals the most app features in which patients were interested. These ICTs, presented as tools that cover these needs, are used as both a source of information and a new communication channel between patients and health professionals. Health professionals should play an essential role in recommending and validating these tools to ensure they are of high quality. Therefore, our results may help in developing possible technological solutions that favor the empowerment of patients and guiding ITCs in routine IMID care.

## Appendix

Multimedia Appendix 1 Questionnaire.

## Abbreviations

ICT: information technology
IMID: immune-mediated inflammatory disease
mHealth: mobile health
