# Supplementary material for: Information and Communication Technologies in Patients With Immune-Mediated Inflammatory Diseases: Cross-sectional Survey
Source: J Med Internet Res. 2022 Sep 13;24(9):e37445. doi: 10.2196/37445 (PMC9516367; doi:10.2196/37445)
Supplement: Multimedia Appendix 1 [file jmir_v24i9e37445_app1.docx]

**QUESTIONNAIRE**

**eHealth and the patient with immune-mediated inflammatory diseases:**

**How do you use new technologies in your health care?**

**All information received is strictly confidential and anonymous.**

The information collected will be useful to develop improvement actions that favor the care of patients with IMID.

**THANK YOU VERY MUCH**

**QUESTIONNAIRE**

This survey intends to know the degree of use of new information and communication technologies in patients with immune-mediated inflammatory diseases (IMID).

Throughout the survey we will ask you a series of questions about the use of mobile technology (internet and mobile applications) related to health.

**SU OPINIÓN NOS INTERESA**

To answer the questions, read the statement carefully and mark with an "X" the most appropriate option:

**BLOCK A: Patient sociodemographic characteristics**

1. Age: _____ years

1. Sex.

Male

Female

1. What is your current occupation?

Works

Retired

Study

Unemployed

Homemaker

1. Level of education.

No education or incomplete primary education

Primary education

Secondary education

Universtity education

1. What type of IMID do you suffer?

Psoriasis

Crohn`disease

Ulcerative Colitis

Rheumatoid arthritis

Juvenile idiopathic arthriti

Spondyloarthritis

Psoriatic arthritis

Other (please specify): _________________

1. How would you rate your overall health?

Very good

Good

Average

Poor

Very poor

### **BLOCK B: ICT Use for Health-Related Information**

1. Which of the following terms have you heard before? *(You can check multiple options at once)*

ITC (Information and communication technologies)

eHealth

Mobile helath or mHealth

Smartphone

App (mobile application)

Wearable

I have not heard any of these terms before

1. Which devices do you use to look for information on the internet? *(You can check multiple options at once)*

Mobile phone

Desktop or laptop computer

Tablet

Televisión with internet (SmartTV)

Smartwatch or smart bracelet (wearable)

Other

I don't use any device

1. How often do you use them to search for information on the Internet?

1. Are you interested in being informed about health issues?

Yes

No

1. Where do you look for health information? *(You can check multiple options at once)*

Health professionals

People close to me (friends, relatives, workmates…)

Newspapers, magazines, pamphlets

Internet

Apps

Other

1. f you use the Internet to search for information about your health, what types of pages do you search? *(You can check multiple options at once)*

Google

Blogs

Youtube

Social networks

Twitter

Facebook

Other

Patient associations

Medical societies

Other

I do not search for medical information on the internet

1. For what purpose do you seek information on health? *(You can check multiple options at once)*

Disease prevention, healthy lifestyle, health care

To find symptoms and learn about potential diseases

To find information about the treatment prescribed by my doctor

To find information about alternative/complementary medicines (herbal products, acupuncture, etc.)

To find information about medical centers or health professionals

To get in contact with other people with health problems like mine

Other

1. Is the health information you find on the internet easy to understand?

Always

Usually

Sometimes

Never

1. Do you trust the health information you find on the internet?

Yes

No

Depends on the website

1. Do you look up information on the internet about your disease or treatment BEFORE going to your doctor’s appointment?

Yes

No

1. Do you look up information on the internet about your disease or treatment AFTER going to your doctor’s appointment?

Yes

Only if I have any doubts

No

**BLOCK C: Use preferences for health apps**

1. Your mobile phone is a Smartphone? (mobile phone with touch screen, which allows the user to connect to the internet, send e-mail, etc.)

Yes

No

1. What do you use your mobile phone for? *(You can check multiple options at once)*

Normal phone use (calls, messages, photos/videos, etc)

Social networks

Schedule planner and alarms

To Access the internet

To use apps

1. Do you have applications (app) related to health?

Sí

No

1. What do you use health-related apps for? *(You can check multiple options at once)*

Prevent diseases, health problems and improve my lifestyle

Obtain information about my disease and its treatments

Manage my appointments with the health center, hospital or health professional

Write down and control my medication

Record and monitor my symptoms

Contact healthcare professionals

Remote monitoring by healthcare professionals

Emotional Support

Other

1. How would you like a health-related app to help you? *(You can check multiple options at once)*

Prevent diseases, health problems and improve my lifestyle

Obtain information about my disease and its treatments

Manage my appointments with the health center, hospital or health professional

Write down and control my medication

Record and monitor my symptoms

Contact healthcare professionals

Remote monitoring by healthcare professionals

Emotional Support

Other

1. How would you like to communicate with your healthcare professional? *(You can check multiple options at once)*

Email

Telephone

App

Social networks

Blog

Website

Videoconference

1. How have you found the health-related apps you use? *(You can check multiple options at once)*

On the recommendation of a family member or friend

Prescription or medical advice

On my own

1. Would you use an app if your health professional recommended it?

No

Yes

1. Would you download a health-related app if you had to pay approximately €2.15 ($2.15)?

No

Probably

Yes

1. Below you can write down any comments you have about the topic discussed:

|  |
| --- |
